# Evolutionary drivers of caching behaviour in corvids

**DOI:** 10.1007/s10071-025-01938-1

**Published:** 2025-02-22

**Authors:** Fran Daw, Bret A. Beheim, Claudia A. F. Wascher

**Affiliations:** 1 Behavioural Ecology Research Group, School of Life Sciences, Anglia Ruskin University, East Road, Cambridge, CB1 1PT UK; 2 Department of Human Behavior, Ecology and Culture, Max Planck Institute for Evolutionary Anthropology, Deutscher Platz 6, 04103 Leipzig, Germany

**Keywords:** Caching, Corvids, Generalist cachers, Socio-ecology, Specialist cachers

## Abstract

Caching has recurrently evolved across a range of animal taxa to withstand fluctuations in food availability and in the context of intraspecific competition. It is widespread in the corvid family, which exhibit considerable interspecific variation in their behavioural and morphological adaptations to caching. However, the evolutionary drivers responsible for this diversity have seldom been explored. The present study systematically reviews the literature on caching behaviour in corvids globally to determine (1) which food caching strategies species have adopted (specialist, generalist or non-cacher) and (2) whether ecological factors affect the occurrence of different strategies, namely (a) climate breadth, (b) trophic niche, (c) habitat breadth, (d) centroid latitude, (e) centroid longitude, (f) breeding system, and (g) body mass. In addition, the ancestral states of caching are reconstructed to assess the evolutionary trajectory of each strategy. Caching strategies were identified in 63 species from 16 genera (out of 128 corvid species and 22 genera). Ancestral state analysis suggested specialist caching as the ancestral state in corvids. Type of caching is associated with distance from equator and by average body mass, with generalist caching concentrated around the equatorial zone and among heavier corvids, while specialist caching occurring more commonly in smaller species found farther from the equator. Although specialist caching most likely was the ancestral state in corvids, both specialist and generalist caching evolved several times independently in the family of corvids. Our results show caching to be widespread in corvids and affected by body size and latitude but ecological factors such as trophic niche and habitat breadth and breeding system, not to be strong drivers shaping caching behaviour.

**Supplementary Information:**

The online version contains supplementary material available at 10.1007/s10071-025-01938-1.

## Introduction

Caching, or hoarding, refers to the storage of food, and occasionally objects, by animals for later use (Vander Wall 1990). From the intricate ‘granaries’ of acorn woodpeckers (*Melanerpes formicivorus,* Oikawa and Pulgarín-R 2019) to the ingenious fruit ripening hoards of tayras (*Eira barbara,* Soley and Alvarado-Díaz 2011). Food storage has recurrently evolved as a behavioural strategy across a wide array of taxa, both vertebrate and invertebrate (Smith and Reichman 1984; Champion De Crespigny et al. 2001; Kim 2010), to strengthen individuals’ resilience to variation in food availability for survival and reproductive success (Vander Wall 1990). Corvids (*Aves*: *Passeriformes*: *Corvidae*), commonly known as the crow family, are of particular interest in regard to caching, largely due to its prevalence and involvement in their spatial and social cognition (Bednekoff and Balda 1996; Clayton et al. 2007), in addition to certain species’ roles as primary seed dispersers (Pesendorfer et al. 2016), including seed dispersal of threatened plant species (Bai et al. 2017). The behaviour shows significant interspecific variation in corvids, especially with concern to the types and amounts of food cached, as well as the duration between its storage and retrieval. For example, western jackdaws (*Coloeus monedula*) seldom cache (Madge and de Juana 2020), whereas Clark’s nutcrackers (*Nucifraga columbiana*) have been estimated to cache between 20,000 to over 100,000 seeds each autumn, upon which they are heavily dependent for overwinter survival and early reproduction (Vander Wall and Balda 1981). Nutcrackers and other cache-dependent species, such as pinyon jays (*Gymnorhinus cyanocephalus*) , often rely on the successful fruiting of specific plant species to the extent that they are forced to irruptively migrate from their breeding ranges during years when yields are low (Brodin 2010; Johnson and Balda 2020) or forego a breeding season (Schaming 2015).

Caching has also resulted in morphological and physiological interspecific variation in corvids. Some species have evolved anatomical specialisations, such as expandable sublingual and oesophageal pouches (Bock and Balda 1973; Vander Wall and Balda 1981) which serve to increase the number of seeds that can be transported to and from cache locations. They have also developed larger hippocampi than less cache-specialised species (Lucas et al. 2004), improving memory performance and enabling accurate cache recovery even months after caches have been made (Balda and Kamil 1992). Other notable examples include adaptations to bill, jaw, tongue morphology and wing length to increase foraging efficiency (Jackowiak et al. 2010; Johnson and Balda 2020). In terms of physiological adaptations to caching, evidence exists that the presence of large quantities of maturing pinyon pine (*Pinus eduli*s) cones stimulate testicular growth in pinyon jays, presumably because they serve as indicators of impending food abundances (Ligon 1978).

While a significant number of studies have investigated the origins of caching in animals (Brodin 2010), very few have examined the potential evolutionary drivers of interspecific variation in species’ behavioural, morphological or physiological adaptations to caching. Vander Wall and Balda (1981) estimated that more specialised adaptations to caching in corvids have arisen from exposure to seasonal food scarcity, resulting in stronger selective pressures to efficiently capitalise on transient resources due to their increased dependence on stored food for survival. Seasonal food scarcity tends to be more pronounced in temperate climates, which has likely contributed significantly to the relative prevalence of food caching taxa at higher latitudes (Smith and Reichman 1984). Climatic effects on food availability might also contribute to the interspecific variation seen in caching. For example, acorn woodpeckers in Central and South America have been reported to cache considerably less than North American populations (Macroberts and Macroberts 1976; Kattan 1988). Climate can also influence how quickly food quality deteriorates after caching, which could affect the adaptiveness of strategies that rely on long-term caches (Sutton et al. 2016). Therefore, species’ geographic distribution might be a significant determining factor regarding the extent of corvids’ specialisation to caching.

The present study reviews the existing literature on caching in corvids to (1) identify the food caching strategies of corvid species worldwide and (2) determine whether socio-ecological factors explain their interspecific variation in food caching. This study also examines evolutionary trajectories of corvids’ different caching strategies via a reconstruction of the ancestral states of caching, to the aim of contributing new insights into the evolutionary divergence of food caching in the corvid phylogeny. Socio-ecological factors estimated to affect cache dependence, namely (a) climate breadth, (b) trophic niche, (c) habitat breadth, (d) centroid latitude, (e) centroid longitude, (f) breeding system, as well as (g) body mass were hypothesised to determine the extent of corvids’ specialisation to food caching. We predict specialist caching to present an adaption to harsh environments and hence depend on centroid latitude and longitude as well as climatic factors. Similarly, caching in corvids often occurs as a means to cope with food competition (Grodzinski and Clayton 2010), hence we expect breeding system to affect caching behaviour and generalist caching to occur more in group living species compared to territorial pairs in order to mitigate food competition. Caching behaviour has previously been shown to be affected by body mass (Price et al. 2000). We expect more generalist cachers in species with larger body mass compared to specialist cachers.

## Methods

### Data collection

In order to identify species’ food caching strategies, the existing literature on corvid foraging ecology and behaviour was systematically reviewed. Scientific and common names were standardized using a combination of the IOC World Bird List version 14.1. (Gill et al. 2024) and the ‘Clements Checklist’ (Clements et al. 2024) and 128 species of corvids were included. Literature searches have been conducted using Google Scholar and Web of Science, using the keywords: caching, cache, hoarding, hoard, storage, storing, store and hiding as well as each species' scientific name (including outdated or synonymous scientific names). Search outputs have been scanned by Fran Daw (FD) for evidence of caching behaviour. Additionally, secondary literature on corvids has been used (Goodwin 1986; Billerman et al. 2022). References of sources used to classify caching behaviour is provided in the online supplementary materials. Species were categorised as either (a) specialist cachers, (b) generalist cachers, (c) unclassified cachers or (d) non-cachers according to the overall extent of their behavioural specialisation to caching, as indicated by recovery rates and duration of caches, caching intensity and whether they show seasonal variation in caching activity. Specialist cachers were characterised as species for which caching activity typically varies seasonally, peaking in response to the abundance of an ephemeral resource of typically nonperishable food items, such as seeds and nuts, for long-term retrieval. In contrast, generalist cachers cache a wide range of foods over short durations with little variation between seasons. A couple of species (unicolored jay, Aphelocoma unicolor; tufted jay, Cyanocorax dickeyi; Iberian magpie, Cyanopica cooki; rufous treepie, *Dendrocitta vagabunda*, Xinjiang ground-jay*, Podoces biddulphi*) were clearly identified as cachers from the literature, however only limited information on context was available and hence they have been categorized as unclassified. Non-cachers were reported to never, or only rarely, cache. Categorisations were graded on the reliability of the sources (1 = low, 2 = medium and 3 = high) by FD, according to their quantity, quality and congruence and whether the behaviours were recorded in wild or captive individuals. Source quality was assessed based on factors such as how many observations were recorded, observer/author confidence and how comprehensively the behaviours were described.

In order to substantiate and expand upon the data obtained from the literature, information on caching behaviour was also collected via an anonymous online survey. The survey was advertised to researchers, animal keeping professionals and other persons with experience observing wild or captive corvids over an extended period. Participants were presented with up to 27 questions concerning the caching behaviours of the species they have experience with, as well as factors that might influence their expression, such as age, food sources and environment. Participants were also asked for information regarding factors that could affect the detection of certain behaviours, such as how regularly species were observed. Participants were familiarised with caching behaviour in corvids beforehand via a short video showing wild ravens (*Corvus corax*) caching acorns, after which they were asked to rate their ability to recognise the behaviour on a Likert scale before proceeding. Participants with experience observing more than one corvid species were invited to submit additional responses. Participants were not limited in the number of responses that could be submitted. The survey was hosted using JISC and distributed via the social media platform X, formerly Twitter, and email. Email recipients were restricted to Species360 member institutions registered as holding at least one corvid species on the Species360 Zoological Information Management System (ZIMS). Responses were accepted for three weeks (November 22 to December 13, 2021). Ethical approval was received from the School Research Ethics Panel at Anglia Ruskin University prior to conducting the survey (A&EB SREP20-04). Survey questions are provided in supplementary file 1.

### Socio-ecology of corvids

Data were collected on several factors relating to corvids’ socio-ecology that were expected to affect specialisation to food caching. These included (a) climate, (b) trophic niche, (c) habitat breadth, (d) centroid latitude, (e) centroid longitude, (f) breeding biology and (g) body mass. Climate data was sourced from the International Union for Conservation of Nature (IUCN) Habitat Classification Scheme 3.1 (HCS) and number of climate categories a species occurs, temperate, tropical, arid and polar was counted (1–4). Species were classified as inhabiting polar and arid climates if they occur in habitats situated above or near polar latitudes (*e.g.* tundra) and those classified as hot, arid, semi-arid, dry and Mediterranean under the IUCN HCS, respectively. In line with IUCN classifications, species occurring in either subtropical or tropical climates were regarded as tropical. For species not currently assessed by the IUCN, classifications for species they consider synonymous (*e.g. Aphelocoma californica* and *insularis*) were used. Habitat breadth was assessed using the HCS, from which species were categorised as either (a) habitat specialists or (b) habitat generalists based on their dependence on certain habitats. Trophic niche (frugivore, granivore, invertivore, omnivore), centroid latitude and longitude as well as body mass have been extracted from AVONET (Tobias et al. 2022) and (Atwood 1980; Benmazouz et al. 2023). Breeding biology (territorial pairs versus groups, which includes family groups, communal and cooperative breeding) was recorded from Birds of the World (Billerman et al. 2022).

### Statistical analysis

All analyses were conducted in R version 4.3.1. (R Core Team 2019). The maximum clade credibility (MCC) tree of corvids from Open Tree of Life (OpenTreeOfLife 2019) was chosen as the foundational phylogeny for the ancestral state reconstruction (ASR) of corvids’ food caching strategies and Bayesian generalized linear mixed-effects model. Two species (*Pica nuttalli,* a generalist cacher and *Pica serica,* caching behaviour not reported) had to be excluded from further statistical analysis as they were missing from the phylogenetic tree. The phylogeny was pruned to exclusively contain species that had been categorised as either specialist, generalist or non-cacher. Including each strategy as a discrete character, the ancestral states of caching for each clade were estimated under equal-rates (ER), symmetrical-rates (SYM), all-rates-different (ARD) custom continuous-time Markov chain (CTMC) models in the R using the package phytools version 1.6.−16 (Revell 2012). We used stochastic character mapping to estimate the number of state changes across the phylogeny, also using an ER model. Akaike information criterion (AIC) values and their corresponding weights were calculated to assess the fit quality of each model. Akaike weights from each model were similar, indicating the strength of evidence supporting each of the three different models being quite similar to one another, hence we have used model averaging in order to consider outcomes of all models.

We employed a Bayesian generalized linear mixed-effects model built with the MCMCglmm package (Hadfield 2010) to determine whether socio-ecological factors affected caching behaviour in corvids. We ran Markov chain Monte Carlo (MCMC) chains for 3,000,000 iterations, thinned by 2000, and employed a burn-in of 2,000,000. All main effects were given normal(0, 0.5) log-odds prior, with a normal(0, 1) log-odds intercept prior. Random effects by genus were given an inverse-Wishart prior with V = 1 and nu = 10. We included phylogenetic relatedness (using the phylogenetic tree shown above) as random factor in these models. Posterior estimates were diagnosed for sampling convergence by the Gelman-Rubin statistic and by visual inspection of chains. Because only three species have been classed as non-cachers, we have focussed statistical analysis on generalist versus specialist cachers. Number of climate categories, trophic niche, habitat breadth, centroid latitude (transformed into absolute values), centroid longitude (transformed into absolute values), body mass and breeding system were included as fixed factors. Four species had to be excluded from statistical analysis because data on body mass, or centroid latitude and longitude was missing (*Aphelocoma woodhouseii, Calocitta colliei, Corvus cornix, Corvus hawaiiensis*), resulting in 50 species (30 generalist, 20 specialist) included in the MCMCglmm analysis. Data and code of the present study are published as online supplementary materials (Supplementary File 2; supplementary File 3).

## Results

**Fig. 1:**
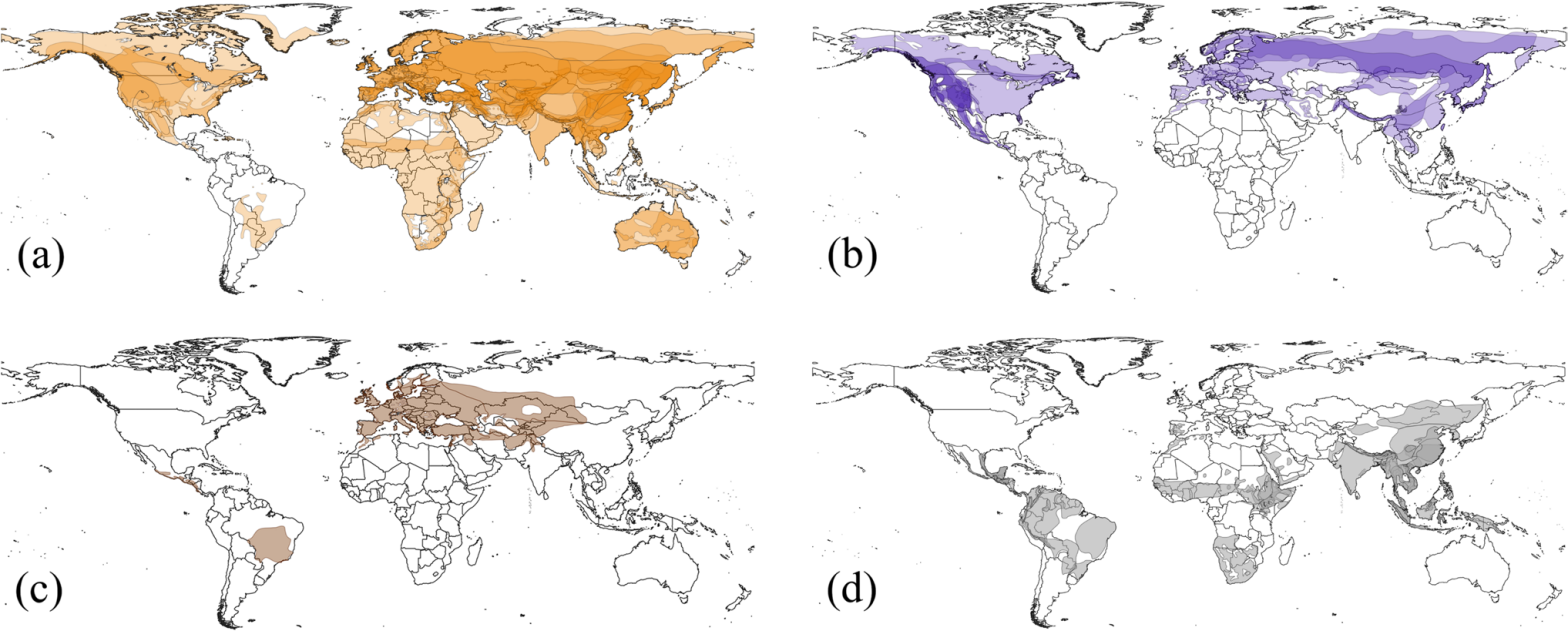
The geographic distribution of corvids according to their food caching strategies, including (**a**) generalist cachers (*n* = 34), (**b**) specialist cachers (*n* = 21) and (**c**) non-cachers (*n* = 3). Also shown are the distributions of (d) unclassified species and those for which no records on caching behaviour were found (*n* = 65). Darker regions indicate where ranges overlap

From the literature search and survey combined, evidence of caching activity (or lack thereof) was found in 63 out of 128 species from 16 out of 22 genera, of which 58 species from 15 genera could be confidently categorised as either specialist cacher (*n* = 21), generalist cacher (*n* = 34) or non-cacher (*n* = 3). Caching had been reported in a further five species (unicolored jay, Aphelocoma unicolor; tufted jay, Cyanocorax dickeyi; Iberian magpie, Cyanopica cooki; rufous treepie, *Dendrocitta vagabunda*, Xinjiang ground-jay*, Podoces biddulphi*), however information was so sparce that they could not be categorized as generalist or specialist cachers. The survey received 57 responses on 23 species, including one species for which caching had not previously been reported (*Cyanocorax colliei)*. Categorisation reliability was graded as high for 38% of species, moderate for 30% and low for 32%. There were 65 species for which no records on caching behaviour were found. Generalist cachers were found to occur on every continent except Antarctica, whereas specialist cachers were absent from the Southern Hemisphere, including South America, Australia and all except the most northerly regions of Africa. Non-cachers were found to occur in South and Central America, Eurasia and northernmost Africa (Fig. 1).

**Fig. 2:**
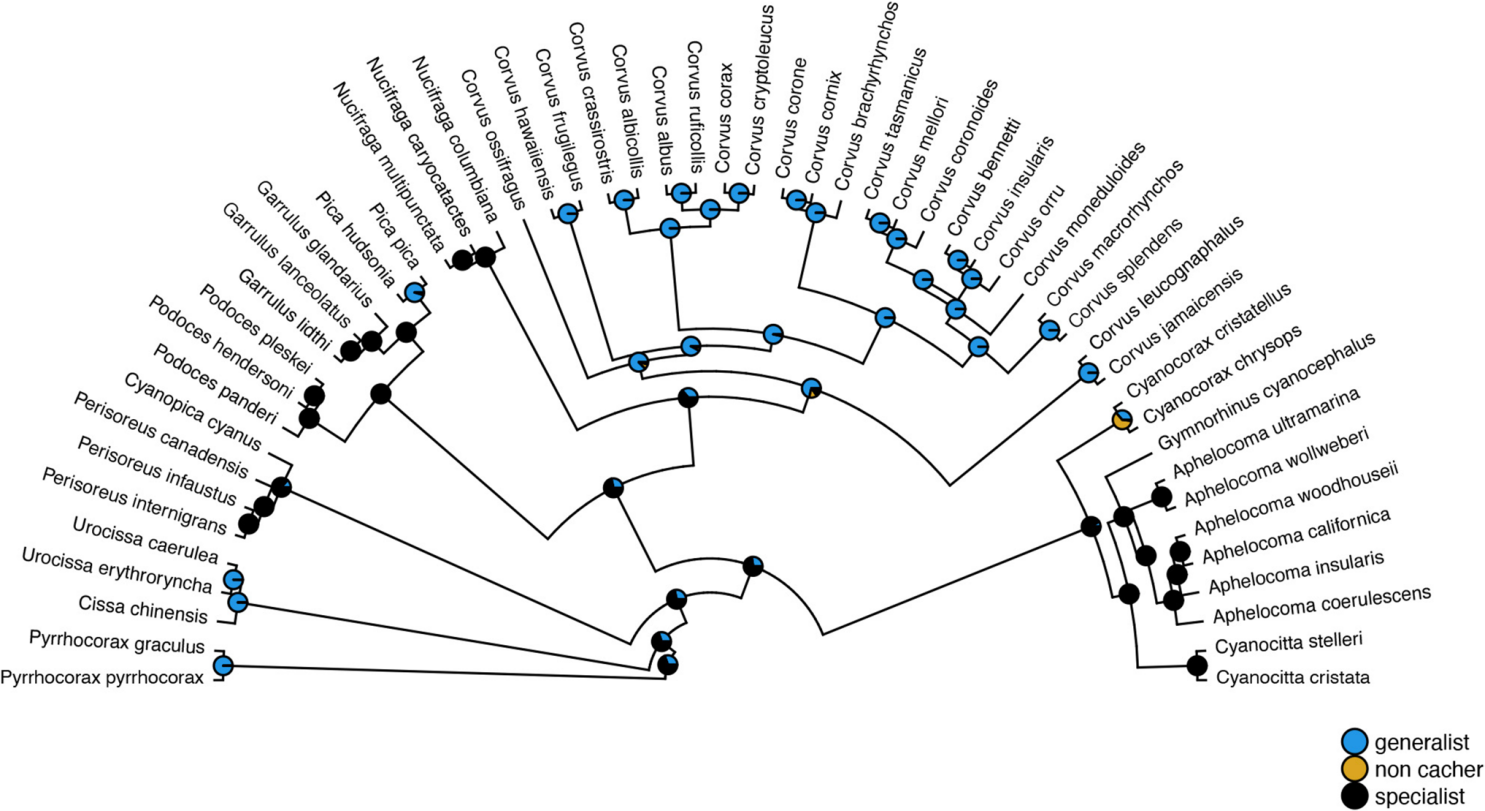
Ancestral state reconstruction of food caching in corvids

Ancestral states estimated under the ARD, ER, and SYM models had similar AIC weights (Table 1), hence we conducted model averaging. Specialist caching was estimated to be the ancestral state of caching in corvids with a (scaled) likelihood of 69%, compared to generalist caching (31%) and non-caching (0%; Fig. 2).

**Table 1:** Log-likelihoods (Log*L*), degrees of freedom (df), Akaike information criterion (AIC) values and their corresponding weights (*w*_*i*_) for ancestral state estimation of food caching in corvids under equal-rates (ER), all-rates-different (ARD), symmetrical-rates (SYM) models

Model	Log(*L*)	df	AIC	*w*_*i*_
ARD	−19.611	6	51.223	0.514
ER	−25.368	1	52.737	0.241
SYM	−23.357	3	52.714	0244

Models listed in descending order of weight

**Fig. 3:**
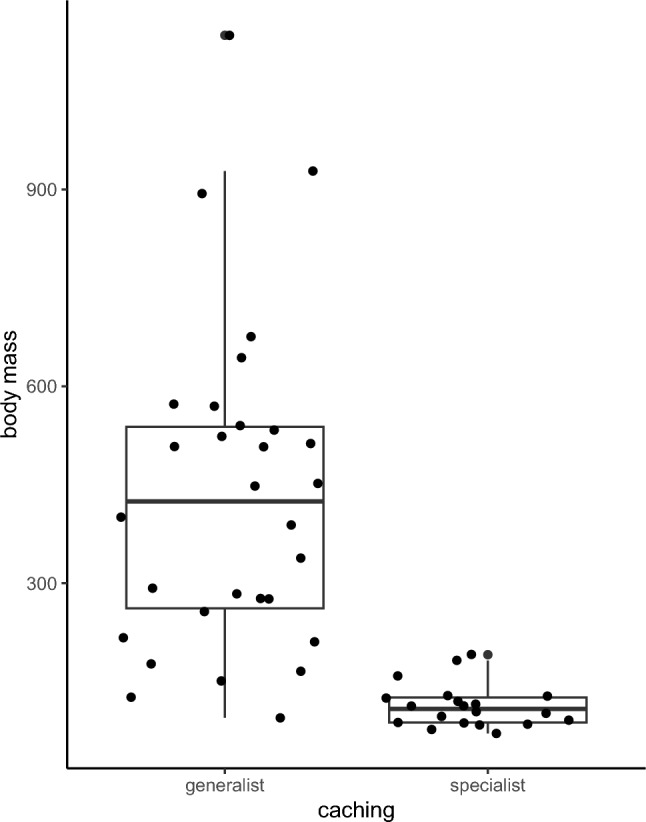
Caching behaviour in relation to body mass (g)

Type of caching is associated with distance from equator and by average body mass, with generalist caching concentrated around the equatorial zone and among heavier corvids, while specialist caching occurring more commonly in smaller species found further from the equator (Fig. 1a and b; Fig. 3). Neither breeding system, nor trophic niche or habitat breadth affected whether a species was a specialist or generalist cacher (Table 2).

**Table 2:** Results of Markov chain Monte Carlo generalized linear mixed-effect model (MCMCglmm)

Parameters	post.mean	l-95% CI	u-95% CI	eff.samp	pMCMC
(Intercept)	−0.028	−1.781	1.992	567.707	0.932
Number of climate categories	−0.089	−1.388	1.399	500	0.912
Breeding system	−0.115	−1.448	1.317	500	0.9
Trophic niche (granivore relative to invertivore)	0.032	−1.131	1.514	500	0.952
Trophic niche (granivore relative to omnivore)	0.0233	−1.397	1.273	437.933	0.940
Habitat breadth (generalist relative to hypergeneralist)	0.083	−1.312	1.383	500	0.868
Habitat breadth (generalist relative to specialist)	−0.16	−1.514	1.358	369.619	0.844
**Centroid latitude**	−**0.763**	−**1.593**	−**0.031**	**51.099**	** < 0.002**
Centroid longitude	−0.236	−0.691	0.131	129.246	0.24
**Body mass**	**0.299**	**0.011**	**0.589**	**26.894**	** < 0.002**

Factors with significant effects (*p* ≤ 0.05) are shown in bold

## Discussion

The present study identified the caching strategies of 63 corvid species from 16 genera, including 34 generalist cachers, 21 specialist cachers, 5 unclassified cachers and 3 non-cachers. Our analysis shows caching in corvids to be concentrated around the equatorial zone and not as proportionally common among species towards either pole. This is unexpected as caching, particularly specialist caching, in many species has evolved to ensure food access during periods of food scarcity and presents an adaptation to unpredictability in food availability, such as seasonality (Štorchová et al. 2010; Sutton et al. 2016). Caching has been shown to improve over-winter survival in several species (collared pika, *Ochotona collaris*: Morrison et al. 2009; mountain chickadees, *Poecile gambeli*: Benedict et al. 2020); red squirrel*, Sciurus vulgaris*: Wauters et al. 1995). Therefore, we would have expected caching, especially specialist caching, to occur more in higher latitudes. We did indeed find smaller species, further away from the equator to be specialist cachers, whereas larger species, located more around the equator, tend to be classed as generalist cachers. In endotherms, organisms with low body masses tend to possess higher per-unit basal metabolic rates than those with higher masses (Schmidt-Nielsen 1997). Consequently, small-bodied species need to consume more food relative to their body mass and, as a result, are more sensitive to food scarcity (Millar and Hickling 1990). Therefore, under the same conditions, specialist cachers’ long-term storage of food might be most advantageous for small corvids, whereas generalist caching might be more adaptive for larger species.

Interestingly, our analysis also highlights species in the global south to be understudied compared to species in the global north. Geographic bias is common in ecology and evolutionary biology research and present a significant barrier to our understanding of evolutionary processes, which needs to be addressed urgently (Martin et al. 2012; Archer et al. 2014; White et al. 2021; Ellison et al. 2021). From the 63 corvid species for which we found information on caching behaviour, we could identify 20 primary sources (papers in peer-reviewed journals and doctoral thesis), 42 records in secondary sources (33: Billerman et al. 2022; 9: Goodwin 1986) and only in one species we relied on responses to our anonymous survey. We have advertised our survey on X (previously Twitter) and have e-mailed institutions (zoos, wildlife parks) registered to hold at least one corvid species. This has certainly biased responses towards people working on captive corvids, rather than field researchers observing corvids in the wild. Broadly, the results of the survey confirmed information from the literature, hence we consider responses as credible, however we also highlight great variation in the quality of information available in the literature. To quantify this, we have assessed the subjective reliability of the information available from low (32% of records), moderate (30% of records) to high (38% of records). This highlights the urgent need for more reports of natural history in the literature.

Our analysis shows specialist caching as the most likely ancestral state in corvids. This is in contrast with a previous study, which described ‘moderate’ caching rather than specialist caching as the ancestral state in corvids (de Kort and Clayton 2006). This difference could occur from a slightly different categorization of caching as well as differences in data availability. De Kort and Clayton (2006) describe caching behaviour in 46 species of corvids from 16 genera (28 moderate, 10 specialist and 1 non-caching species), which is 17 species less than we included in our analysis. Three species described as ‘moderate cachers’ by de Kort and Clayton (2006) have been described as specialist cachers in our study (*Aphelocoma californica:* Curry et al. 2020*; Aphelocoma coerulescens: Woolfenden and Fitzpatrick 2020 *and *Aphelocoma ultramarina:* Kamil et al. 1994*)*. In mammals, larder hoarding has been described as the ancestral state of food hoarding, compared to scatter hoarding (Mahoney and Pasch 2024). Importantly, our ancestral state analysis indicates that both generalist and specialist caching have independently evolved multiple times in corvids, raising the question of which socio-ecological factors facilitate shaping the behaviour.

In our analysis caching behaviour was not affected by habitat breadth, number of climate categories, trophic niche and breeding system. This is surprising, as we would have expected these factors to shape caching behaviour in corvids. For example, we would have expected species which are more generalists in terms of their habitat breadth, to be less dependent on the seed productivity of specific plant species and are therefore likely capable of occupying a significantly greater number and range of habitats and different climate categories. Similarly, in terms trophic niche, we expected a link between food perishability and caching behaviour. Indeed, we observed two granivore species to be specialist cachers (*Nucifraga caryocatactes* and *Nucifraga multipunctata*) and three invertivore species (*Cyanocorax chrysops*, *Pyrrhocorax graculus* and *Pyrrhocorax pyrrhocorax*) to be generalist cachers. The link between granivory diet and specialist caching as well as invertivory and generalist caching is expected due to food perishability, with invertebrates being much more perishable compared to seeds and nuts, hence less appropriate for specialist caching. Some specialist cachers are able to mitigate the amplifying effects of certain environmental conditions on food perishability by being selective about where they cache, such as spotted nutcrackers (*Nucifraga caryocatactes*), which have been seen to preferentially cache seeds at sites that slow their degradation (Neuschulz et al. 2015). Furthermore, the specialist-caching Florida scrub jays (*Aphelocoma coerulescens*) are able to learn the rates at which different food types degrade, as well as remember the location and relative time that a given food item was cached. They can then use this information to prioritise retrieving caches containing foods that degrade fastest (Clayton and Dickinson 1999). Additionally, black-capped chickadees (*Poecile atricapillus*) learn to avoid caching at sites where previously cached food items had been lost (Hampton and Sherry 1994). Evidently, caching in corvids is a highly flexible behaviour that individuals can adapt to maximise the longevity and quality (and therefore value) of caches, further demonstrating that both generalist and specialist caching can confer fitness benefits under many different environmental conditions. Flexibility in caching has also been shown in other species, for example European beavers (*Castor fiber*) show a certain level of behavioural plasticity in caching behaviour, depending on their geographic distribution (Busher et al. 2020) and consistent individual differences in caching behaviour have been described in Mongolian gerbils (*Meriones unguiculatus*, Gan et al. 2024).

Another factor which is expected to strongly shape caching behaviour in corvids is conspecific competition (Grodzinski and Clayton 2010). In many corvid species, caching food for later can increase the amount of an ephemeral resource that an individual can acquire (Vander Wall and Balda 1981), however caches might be lost to pilfering conspecifics (Shaw and Clayton 2012) resulting in the evolution of several cache protection strategies (Dally et al. 2006). In the present study, caching was not affected whether corvid species were classed as territorial pairs or group breeding (including family groups, communal nests and cooperative breeders). Previously, cache protection strategies also have been shown in less social species (Clary and Kelly 2011). In future, it would be interesting to further investigate the impact of social interaction patterns, independent of social system on caching behaviour.

In conclusion, in the present study we provide a comprehensive overview of the geographical, morphological, social and ecological factors shaping the evolution of food caching behaviour in corvids. We describe body mass and centroid latitude to affect specialist versus generalist caching, whereas most other ecological factors, such as diet or habitat specialism, or breeding system not to affect caching behaviour. The present study demonstrates that the adaptiveness of corvids’ different caching strategies can be maintained across a wide range of environments.

## Supplementary Information

Below is the link to the electronic supplementary material.

Supplementary file1 (PDF 105 KB)

Supplementary file2 (CSV 94 KB)

Supplementary file3 (R 10 KB)

## Data Availability

Data and analysis code is published as online supplementary materials.
