# Correction: Evolutionary drivers of caching behaviour in corvids

**DOI:** 10.1007/s10071-025-01989-4

**Published:** 2025-07-25

**Authors:** Fran Daw, Bret A. Beheim, Claudia A. F. Wascher

**Affiliations:** 1 Behavioural Ecology Research Group, School of Life Sciences, Anglia Ruskin University, East Road, Cambridge, CB1 1PT UK; 2 Department of Human Behavior, Ecology and Culture, Max Planck Institute for Evolutionary Anthropology, Deutscher Platz 6, 04103 Leipzig, Germany

**Correction: Animal Cognition (2025) 28:17**

10.1007/s10071-025-01938-1

In this article the ‘Conflict of Interest’ statement was missing and should have been “C.A.F.W. received funding from Anglia Ruskin University, and B.A.B. received funding from the Max Planck Institute for Evolutionary Anthropology. F.D. didn’t receive any funding”. The original article has been corrected.

