# Supplementary material for: Evolutionary drivers of caching behaviour in corvids
Source: Anim Cogn. 2025 Feb 22;28(1):17. doi: 10.1007/s10071-025-01938-1 (PMC11846726; doi:10.1007/s10071-025-01938-1)
Supplement: Supplementary file 1 — Supplementary file1 (PDF 105 KB) [file 10071_2025_1938_MOESM1_ESM.pdf]

Caching behaviour survey DRAFT

Introduction

Hello. My name is Fran Daw and I am a third-year zoology undergraduate student at the Anglia Ruskin University School of Life Sciences (Cambridge, UK) conducting research into caching behaviour in corvids (Corvidae) for my dissertation. In order to obtain the information I need for my research, I am reaching out to professional animal keepers and caretakers from zoos, parks, sanctuaries and other animal collections worldwide to ask about any caching related observations they have made while keeping corvids in captivity. Studies on caching behaviour in corvids have historically been limited to a fraction of the 129 species comprising the family, with some species being excluded from the literature entirely. My project aims to create a more inclusive and comprehensive picture of caching behaviour in the corvid family to provide insight into how and why caching differs between species. The observations you share here will be invaluable to my research. **You are still eligible to participate even if you have never observed caching behaviour before.**

You will be presented with up to 26 questions, which should take no more than 10 minutes to complete. Responses are anonymous and you may choose to leave at any time. Responses will only be recorded once you select Finish at the end of the survey. Your data will be used for this study only and will be held for no longer than one year.

Thank you for your help!

https://www.jisc.ac.uk/website/privacy-notice

Age Confirmation Question

You must be at least 18 to take part in this survey. Please confirm your age below ✱ *Required*

- I am 18 or over
- I am under 18

Consent Question

Do you agree to participate in this survey? ✱ *Required*

- ○ Accept
- ○ Decline

What is caching?

In order to accurately respond to the questions this survey contains, participants should understand what defines caching in corvids. The term caching refers to behaviour where food, and occasionally other items, are stored for later use, e.g., when resources become scarce. The sites where items are stored are termed *caches.* Corvids typically cache using their bills to push items underneath substrate or objects, which may then be covered with foliage or other items to conceal them from others. Some species may also use sites above ground to cache items, such as tree cavities.

If you are unfamiliar with caching behaviour in corvids, please watch the video below showing common ravens (Corvus corax) caching acorns before proceeding to the next section.

Screening Question

Do you, or have you ever, cared for one or more species of corvid (Corvidae) in captivity? ✱ *Required*

- Yes
- No

Screening Question 2

Did the individuals kept have year-round access to substrates suitable for cache formation (e.g. sand, soil)? ✱ *Required*

- Yes
- No

If Yes, please list the substrates the individuals had access to below

Which species have you cared for? Please enter the common and scientific name below. If you have experience with more than one species, please submit a separate response

Common name ✱ *Required*

Scientific name ✱ *Required*

Please name the country and region (e.g. state, province) where this species was kept. If you have cared for this species at more than one location, please answer this and all subsequent questions using your experiences from the location where you spent the longest with this species ✱ *Required*

How long have you cared for this species? ✱ *Required*

- Less than 1 year
- 1-3 years
- Over 3 years

How many individuals of this species have you cared for? ✱ *Required*

Which sex? Select all that apply

- Male
- Female
- Unsure

What age? Select all that apply

- Adult
- Juvenile
- Unsure

Please briefly describe the duties you performed for while caring for this species (e.g. feeding, providing enrichment, administering medication) ✱ *Required*

Routed Question

Have you ever observed this species caching food or other objects? ✱ *Required*

- Yes
- No
- Unsure

If Yes, approximately how many individuals of this species have you observed caching?

Time

In an average week, how many days did you spend caring for this species? ✱ *Required*

In a typical day, how much time did you spend with this species? ✱ *Required*

- Less than 1 hour
- 1-3 hours
- 3-5 hours
- 5-8 hours
- More than 8 hours

Behaviour

In a typical year, how often did you observe this species caching food or other items? ✱ *Required*

Please don't select more than 1 answer(s) per row.

Please select at least 1 answer(s).

	+4	+3	+2	+1	Multiple times per month	-1	-2	-3	-4	
Multiple times per day	☐	☐	☐	☐	☐	☐	☐	☐	☐	I have only observed caching once

How long did this species typically take to retrieve their caches? ✱ *Required*

- ○ Less than a day
- ○ Several days
- ○ One week to a month
- ○ Several months
- ○ Unsure

Did caching frequency differ between seasons? ✱ *Required*

- ○ Yes
- ○ No
- ○ Unsure

If the participant observed caching frequency differing between seasons

In which season(s) was caching most frequent? ✱ *Required*

During this season, which item(s) did this species cache most often? ✱ *Required*

- ☐ Meat
- ☐ Fruit
- ☐ Nuts/Seeds
- ☐ Anthropogenic foods
- ☐ Other foods
- ☐ Non-food objects
- ☐ Unsure

Items

What food types did you typically include in this species' diet? Select all that apply ✱ *Required*

- [ ] Meat
- [ ] Fruit
- [ ] Nuts/Seeds
- [ ] Formulated (e.g. pellets)
- [ ] Other

Which items have you seen this species cache? ✱ *Required*

- [ ] Meat
- [ ] Fruit
- [ ] Nuts/Seeds
- [ ] Formulated foods
- [ ] Anthropogenic foods
- [ ] Other foods
- [ ] Non-food objects
- [ ] Unsure

In a typical year, what item(s) does this species cache most often? ✱ *Required*

- [ ] Meat
- [ ] Fruit
- [ ] Nuts/Seeds
- [ ] Formulated foods
- [ ] Anthropogenic foods
- [ ] Other foods
- [ ] Non-food objects
- [ ] Unsure

What was the highest number of caches made by this species within a day? ✱ *Required*

- ◯ 30+
- ◯ 10-30
- ◯ 5-10
- ◯ 1-5

- Unsure
- Not applicable

If the participant was unsure/did not observe their species caching

Based on the amount of time you have spent observing this species, how confident are you that you would have observed caching behaviour at some point? ✱ *Required*

Please don't select more than 1 answer(s) per row.

Please select at least 1 answer(s).

	+3	+2	+1	0	-1	-2	-3	
Very confident	☐	☐	☐	☐	☐	☐	☐	Very unconfident

Are there any additional comments or observations that you feel would be useful to share?

Thank you for your participation!

Your responses have been recorded. If you have experience caring for another species and would like to submit another response, you can do so via the link below.
[Survey link]

Key for selection options

12 - In an average week, how many days did you spend caring for this species?

1
2
3
4
5
6
7
